# Clinical symptoms, pathogenesis and postoperative course of non-specific constrictive pericarditis with dumbbell-shaped heart

**DOI:** 10.3205/dgkh000463

**Published:** 2024-02-21

**Authors:** Feridoun Sabzi, Reza Faraji, Saeed Khoshnood

**Affiliations:** 1 Department of General Surgery, School of Medicine, Kermanshah University of Medical Sciences, Kermanshah, Iran; 2 Tuberculosis and Lung Diseases Research Center, Ilam University of Medical Sciences, Ilam, Iran; 3 Clinical Microbiology Research Center, Ilam University of Medical Sciences, Ilam, Iran

**Keywords:** constrictive pericarditis, tuberculosis

## Abstract

Non-specific chronic constrictive pericarditis is a rare and debilitating chronic infection in developed countries and its rapid diagnosis and treatment has not affected its outcome and complication.

A 15-year-old male, well nourished, negative HIV test, and without a history of previous pulmonary tuberculosis, was admitted to our hospital for exertional dyspnea (New York Heart Association, NYHA, functional class II). Our patient had had no pulmonary tuberculosis during childhood, had received anti-tuberculosis treatment, and was referred to our center for further surgical pericardiectomy.

## Abstract (de)

Die unspezifische konstriktive Perikarditis ist eine seltene und belastende chronische Infektion in Industrieländern. Ihre rasche Diagnose und Behandlung hat sich jedoch nicht auf ihre Folgen und Komplikationen ausgewirkt. Ein 15-jähriger Mann, gut ernährt, mit negativem HIV-Test und ohne Vorgeschichte einer Lungentuberkulose, wurde wegen Belastungsdyspnoe (New York Heart Association; NYHA, Funktionsklasse II) in unser Krankenhaus eingeliefert. Der Patient hatte in seiner Kindheit keine Lungentuberkulose, erhielt eine Anti-Tuberkulose-Behandlung und wurde zur weiteren chirurgischen Perikardektomie in unser Zentrum überwiesen.

## Introduction

Tuberculous pericarditis often results in severe calcification of the pericardium, the atrio-ventricular groove, and posterior or anterior mitral or tricuspid valves [1]. In the present case, however, tuberculous pericarditis was considered, but other types of pericarditis could not be completely ruled out. It was possible that chronic non-specific constrictive pericarditis had healed and showed no tubercle bacilli in culture. The constricted band observed in the case presented here consisted of a thick layer of calcified tissue within the pericardial cavity. The patient’s previous disease history, clinical course, and the pathological examinations of the resected pericardium showed non-specific etiology for its formation and calcification of the pericardium. 

## Case report

**Figure 1:**
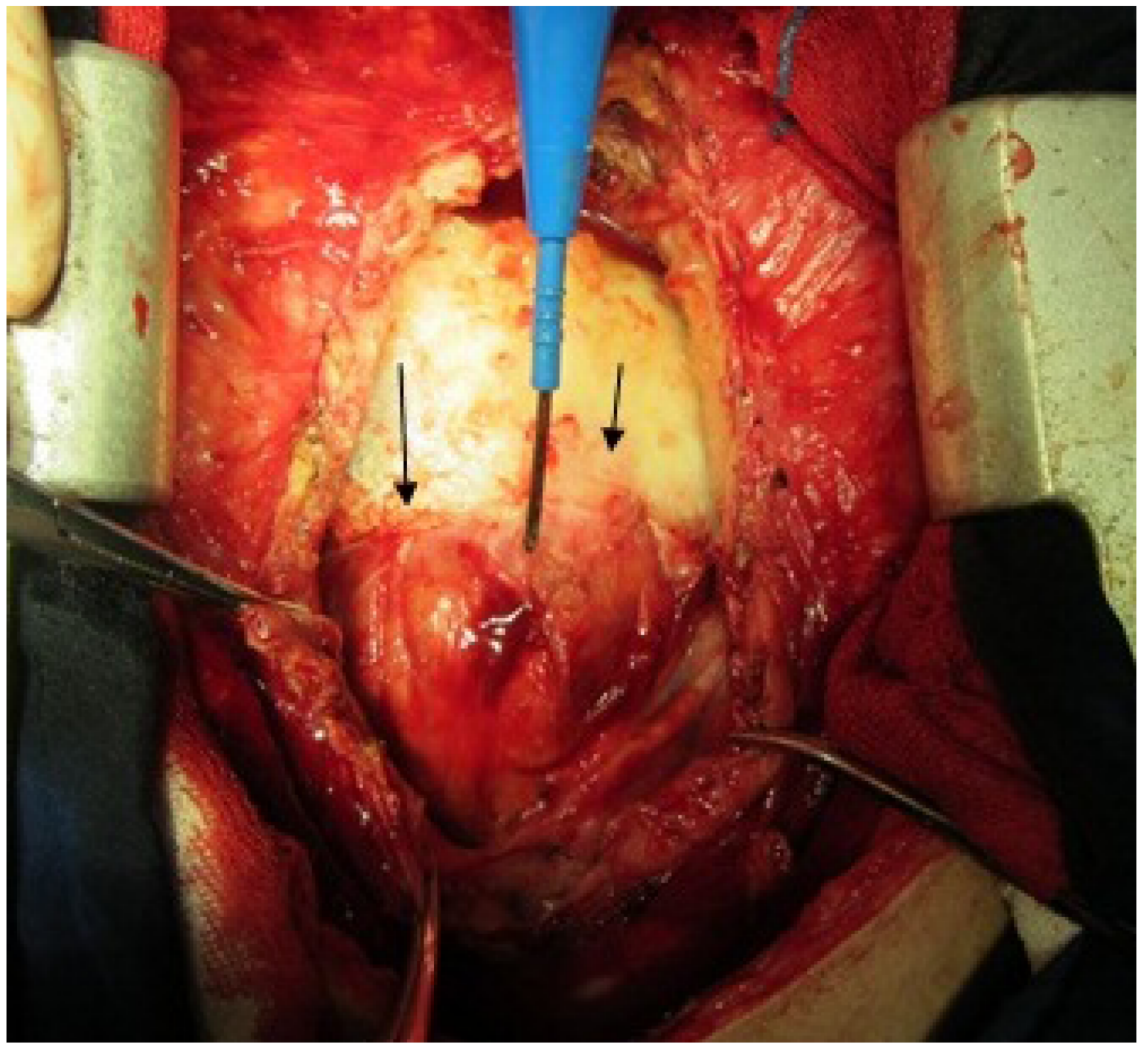
Border between right ventricle outflow tract and large pulmonary artery (long straight arrow). Short arrow shows border between aorta and right ventricle.

**Figure 2:**
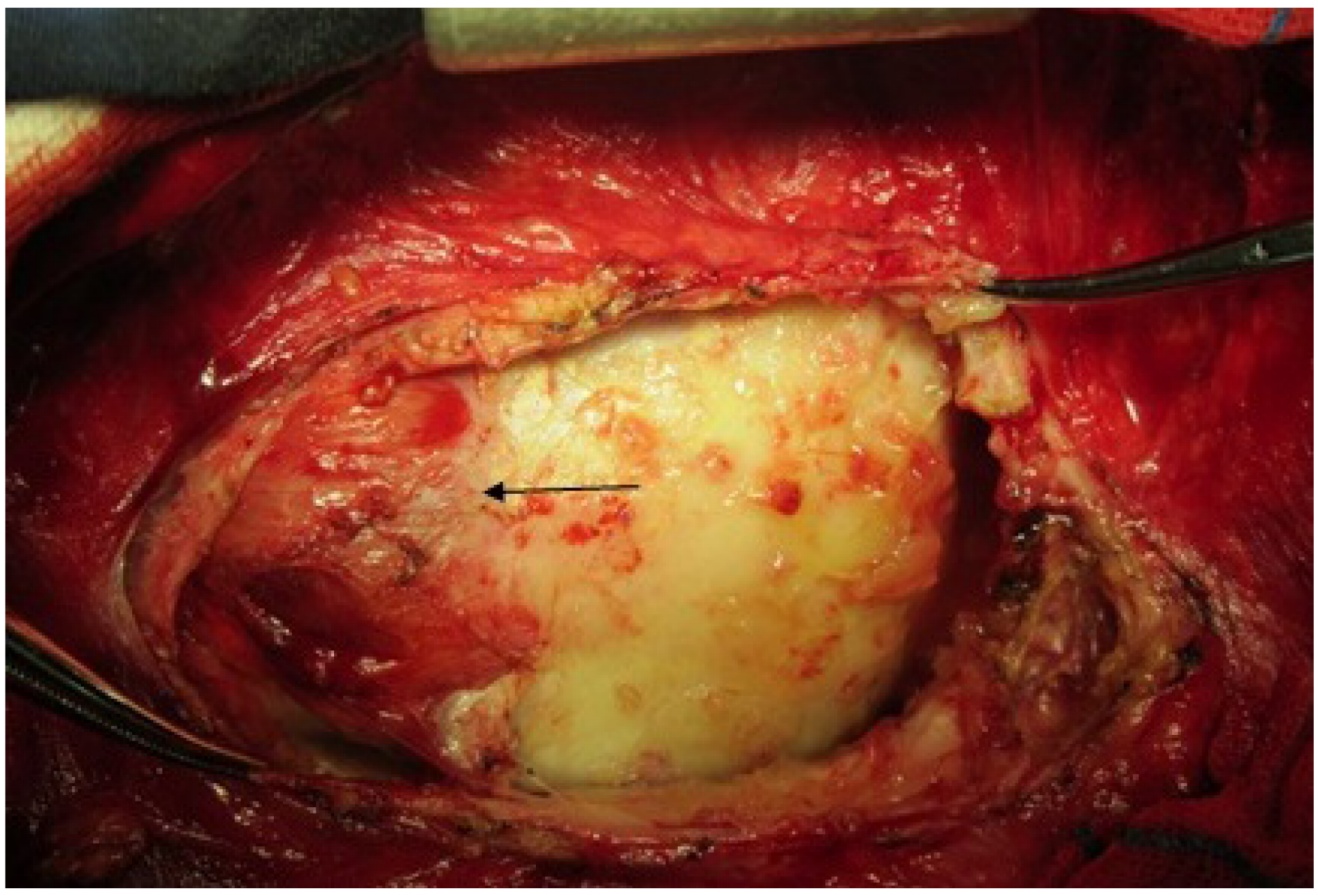
Constrictive band in RVOT (arrow)

**Figure 3:**
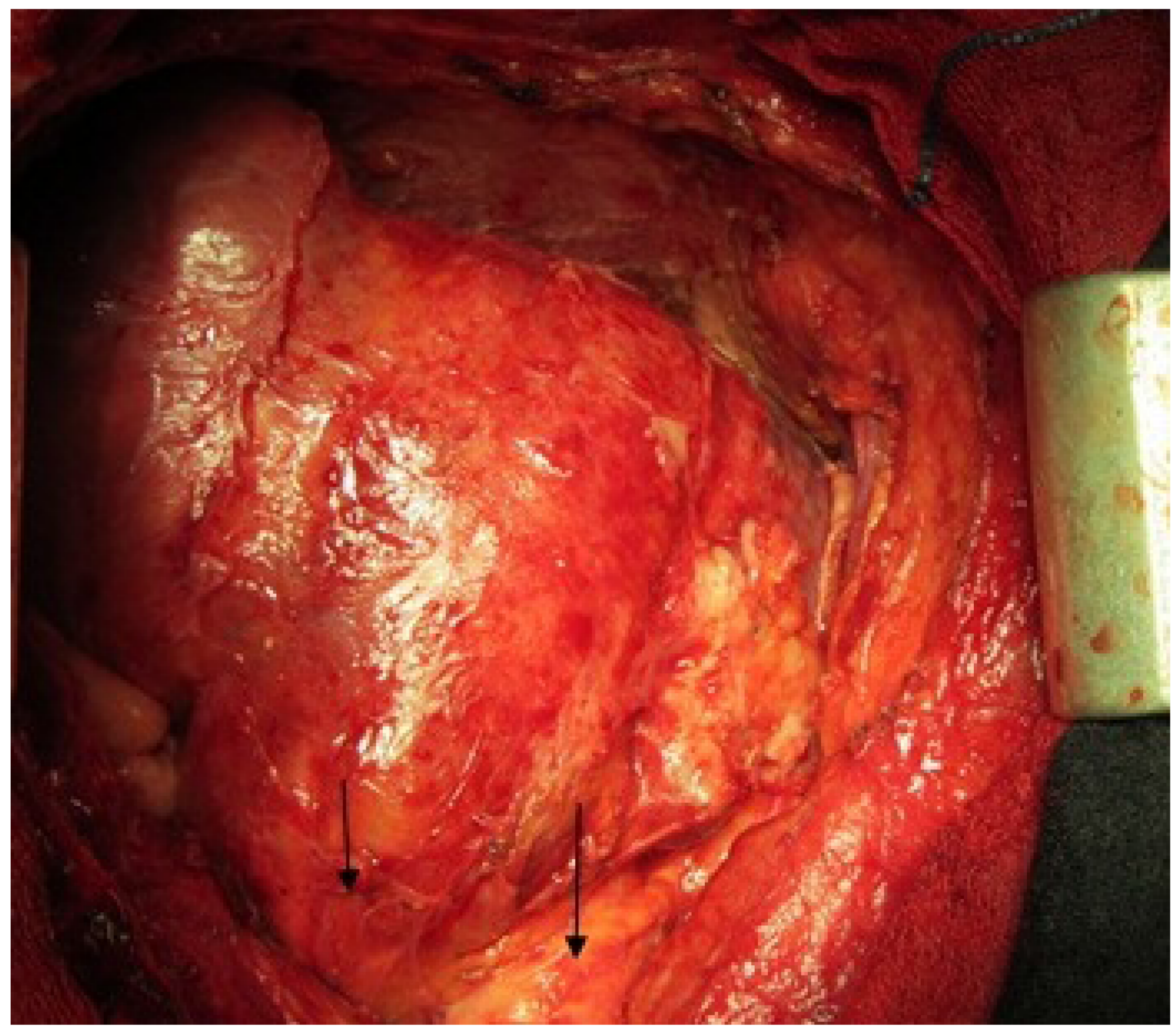
Postpericardiectomy view of heart (small arrow shows RVOT and long arrow shows aorta)

**Figure 4:**
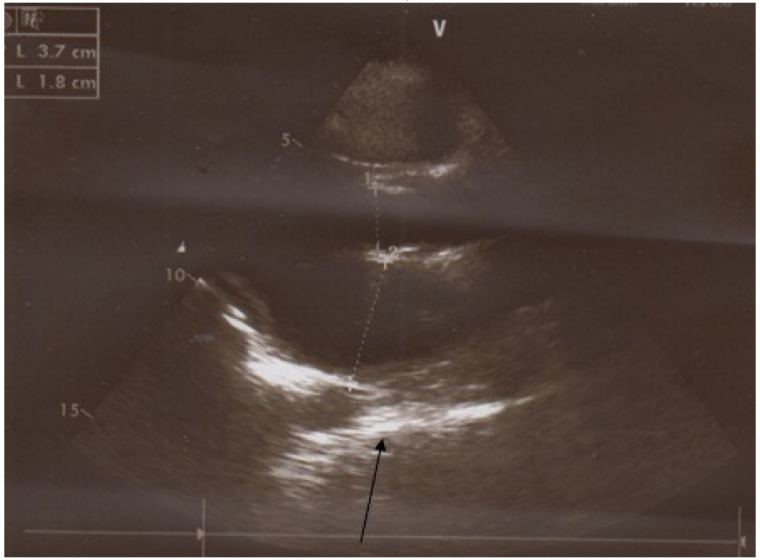
Constrictive band

**Figure 5:**
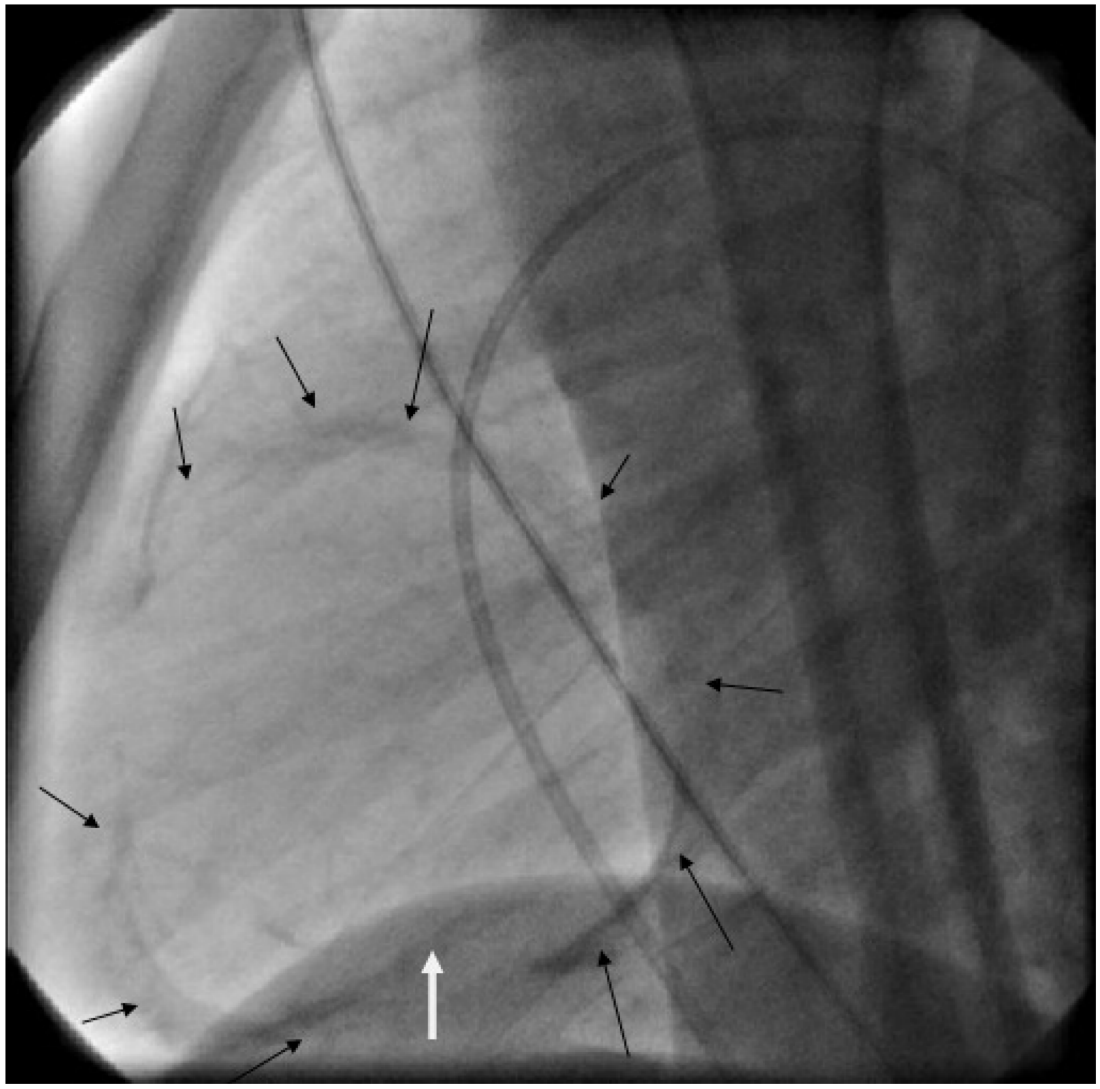
Circumferential calcified band in lateral chest radiograph

A 15-year-old male patient presented to our hospital with pitting edema of the lower extremities, ascites and dyspnea on exertion for 2 months. There was no chest discomfort, orthopnea or paroxysmal nocturnal dyspnea. He did not have symptoms of cough, fever, or weight loss. Physical examination revealed edematous legs but with elevated jugular venous pressure, positive Kussmaul’s sign, and hepatomegaly. No pleural effusion, no patchy infiltration at lung field, and mild cardiomegaly were noted on the chest radiograph. After medical treatment, the abdominal distension and peripheral edema improved, but the patient still complained during hospitalization of mild dyspnea on exertion. Echocardiography revealed a calcified pericardium, especially over the right atrium, superior vena cava (IVC), superior vena cava (SVC) and right ventricular outflow tract (RVOT), and a small amount of pericardial effusion. The left ventricular wall motion was severely depressed. However, decreased RVOT wall motion and a distended inferior vena cava with blunted respiratory variation were observed. Under the impression of right heart failure due to constrictive pericarditis, the patient underwent cardiac catheterization. The hemodynamic studies (Figure 1 (Fig. 1), Figure 2 (Fig. 2), Figure 3 (Fig. 3), Figure 4 (Fig. 4), and Figure 5 (Fig. 5)) showed a right atrium pressure of 20 mmHg, pulmonary capillary wedge pressure of 22 mmHg, prominent *x* and *y* descent in right atrial pressure tracings, and a dip-and-plateau pattern in the right ventricular diastolic pressure tracings. There was equalization of the end-diastolic pressures between right and left ventricles (18 mmHg). Because of the diagnosis of constrictive pericarditis complicated with right heart failure, surgery for pericardial stripping was performed. Operative findings included markedly thickened pericardium on the diaphragmatic surface of the right ventricle, patchy constriction of the anterior surface of right ventricle (RV), IVC, SVC, and a semilunar constricted band that extended from the tricuspid ring anteriorly to infundibulum mending in the mitral annulus. The postoperative course was uneventful, with improvement of heart function. Pathology of the removed pericardium showed non-specific chronic pericarditis with no caseous necrosis.

## Discussion

Fauci et al. [2] showed that chronic non-specific constrictive pericarditis results when the healing of an acute fibrinous or serofibrinous pericarditis or the resorption of a chronic pericardial effusion is followed by obliteration of the pericardial cavity with the formation of granulation tissue. Kothari et al. [3] revealed that constrictive pericarditis is a serious sequel of tuberculous pericarditis, developing in approximately 70% of the patients despite administration of antitubercular drugs and prednisolone. Retrograde lymphatic spread of *Mycobacterium* *tuberculosis* from peritracheal, peribronchial or mediastinal lymph nodes or hematogenous spread from primary tuberculous infection may result in pericardial involvement. Protein antigens of the bacillus induce delayed hypersensitivity responses, stimulating lymphocytes to release lymphokines that activate macrophages and influence granuloma formation. The immune response to the viable acid-fast bacilli penetrating the pericardium is responsible for the morbidity associated with tuberculous pericarditis. Yang et al. [4] showed that constrictive pericarditis is a process of chronic fibrous thickening of the pericardium, which is frequently accompanied by calcification, and prevents the diastolic filling of the heart, reducing venous return and lowering output. Maisch et al. [5] revealed that pericardial constriction results from chronic inflammation of the pericardium, leading to pericardial scarring, thickening, fibrosis, and calcification. Chowdhury et al. [6] described an unusual case of a young man presenting with calcified constrictive pericarditis, and revealed the presence of a thickened pericardium and a thickened calcific constrictive band around the atrioventricular groove posteriorly and over the infundibulum anteriorly. Intraoperatively, the band caused the heart to have a “dumbbell” appearance. A pericardiectomy was performed along with excision of the constricting band. The patient had an uneventful recovery. Iseki et al. [7] reported a 62-year-old man with increasing palpitations. Magnetic resonance imaging revealed compression of the right ventricle by a tumor. At the time of cardiac catheterization, the coronary arteries were found not to supply blood of the mass in the tumor, and no dip-and-plateau pattern was seen in the right ventricular pressure measurements. At the time of surgery, the tumor mass was found to be a focal calcified thickening of the pericardium containing only pus. The thickening resembled an oval pericardial tumor. Microbiological examination of the pus revealed *Propionibacterium* *acnes*. In a study by Bashi et al. [8], a transthoracic echocardiogram showed a thickened pericardium localized throughout the left ventricle, impairing diastolic filling. Doppler waveforms were suggestive of localized constrictive pericarditis. A CT scan of the chest confirmed the presence of unilateral pleural effusion with thickened pericardium surrounding the left ventricle. Liu et al. [9] reported a case in which during surgery, severe calcification of the pericardium associated with circumferential encasement of the ventricles by dense masses was found. Extensive pericardiectomy was performed with mass excision. Histopathology revealed the masses to be non-specific pericarditis. Non-specific constrictive pericarditis is not uncommon; however, non-specific pericarditis leads to compression of the LV, and heart failure is rare. Akdemir et al. [10] reported a case of localized calcified constrictive pericarditis masquerading as a basal aneurysm. The treatment of non-specific pericardial constriction initially involves the use of conventional antituberculous drugs for 6 months and pericardiectomy for persistent constriction during the drug therapy. Pericardiectomy is recommended if the patient's condition is hemodynamically static or deteriorates after 4 to 8 weeks of medical therapy. If, however, the disease is associated with pericardial calcification, a marker of chronic disease, surgery should be performed earlier. The risk of death after pericardiectomy in patients with non-specific constrictive pericarditis ranges from 3% to 16%.

## Notes

### Author’s ORCID

Reza Faraji: https://orcid.org/0000-0002-5973-7301

### Competing interests

The authors declare that they have no competing interests.
